# P-89. Daptomycin Compared to Vancomycin for the Treatment of Osteomyelitis: a national VA cohort study

**DOI:** 10.1093/ofid/ofae631.296

**Published:** 2025-01-29

**Authors:** Ryan P Moenster, Travis W Linneman, Daniel Eaton

**Affiliations:** UHSP/VA St. Louis HCS, St. Louis, Missouri; VA St. Louis HCS, St. Louis, Missouri; VA St. Louis HCS, St. Louis, Missouri

## Abstract

**Background:**

Daptomycin (DPT) has been used as an alternative to vancomycin (VCM) for the treatment of osteomyelitis (OM), but few studies have compared their efficacy in this infection.

**Methods:**

This was a retrospective evaluation of all patients within the Veteran’s Affairs (VA) Health Care System between the ages of 18 and 89 who were treated for OM with at least 21-days of either intravenous (IV) VCM or DPT between 1 January 2009 and 1 January 2020. The diagnosis of OM was identified by a primary ICD-9 (730.0-730.99) or ICD-10 (M86.0-M86.9) discharge code around the time of VCM or DPT administration. Additionally, records were verified to ensure no patient had received more than 6 days of the other therapy.

The primary outcome was clinical failure, which was a composite of retreating with antibiotics for at least 14 days within the 6-months after initial treatment was completed, surgical procedures related to OM in the 6-months after treatment was completed, or all cause 30-day readmission while on antibiotic therapy for the incident case of OM.

Two additional, nested cohorts were analyzed. A cohort matched based on age, race, sex, body mass index, and Charlson Comorbidly Index, and a cohort analyzed using average effect on treatment (ATT).

**Results:**

Five thousand, four hundred twenty-six patients were included (4777 VCM, 649 DPT). The primary outcome of composite clinical failure occurred in 63.4% (412) of DPT patients and 57.2% (2731) of VCM patients (*p* < 0.001). In the adjusted cohort, DPT was not associated with failure (OR 1.173, 95% CI 0.978-1.407), but overall retreatment (1-180 days after completion of initial therapy) and early retreatment (1-25 days after completion of initial therapy) were more likely in DPT treated patients (OR 1.23 (95% CI 1.026-1.475) and OR 1.242 (95% CI 1.092-1.414), respectively). In the ATT analysis composite failure was not associated with DPT, but overall retreatment and early retreatment were (OR 1.263 (95% CI 1.014-1.574) and OR 1.247 (95% CI 1.068-1.455), respectively).

**Conclusion:**

In this large, retrospective cohort of veteran’s receiving treatment for OM more patients receiving DPT experienced composite failure compared to those treated with VCM. The difference appears driven by DPT failure within the first 25 days after treatment completion.

**Disclosures:**

**All Authors**: No reported disclosures

